# The Amendment Bill Now before Parliament

**Published:** 1917-12-01

**Authors:** 


### NATIONAL INSURANCE ACT DEVELOPMENTS.

### The Amendment Bill Now Before Parliament.

A Bill * to amend and simplify the National Health Insurance Acts has, at last, after inordinately long delay, been presented to Parliament. Copies in print became available on November 10, and a full debate on the second reading took place on November 23, when the Bill duly passed that stage without a division, and was passed forward for consideration in Committee.

In spite of the fact that the Bill contains much technical matter, and that many of its provisions cannot be understood except by reference to the principal Act, quite an unusually large number of members from each section of the House took part in the discussion.

### General Outline of the Bill.

It may be said at once that the Bill is without question the most important legislative measure in connection with national insurance that has been before Parliament since the original Bill of 1911. Its objects may be described as twofold, corresponding to the two main sections into which the Bill is separated. Part I. is designed to rectify, or at least to mitigate, the defects in the original financial estimates, particularly in regard to the insurance of women; while Part II. deals with matters calculated to simplify the practical administration of the system. Incidentally, it may be mentioned that a new benefit is introduced in Part II., to be known as "marriage benefit," and that, with a view to making the Act more effective in the prevention of sickness, considerably wider powers are given whereby it will be possible to investigate the causes of excessive sickness, and bring home to the persons or authorities concerned the necessity for the provision of proper safeguards by charging them with the cost of the excessive payments on account of sickness benefits that have been made by approved societies on account of their past negligence.

### The Financial Provisions.

It will be remembered that the benefits given under the original scheme were estimated to be equivalent in value, after making due allowance for administrative expenses, to the joint contributions of the employer and employee, on the assumption that the age at entry into insurance was sixteen years. But beyond these joint contributions the State was to contribute towards the benefits. Seeing, however, that all persons who entered insurance as employed contributors within the first year after the Act came into operation were given the advantage of the right to receive benefits at the rate applicable to age sixteen, irrespective of their ages at entry, it became necessary for certain book credits—technically termed "reserve values"—to be credited to approved societies to enable them to discharge their obligations in respect of such persons. The scheme then provided that the Insurance Commissioners should retain a portion of each contribution paid in order to liquidate these book credits, and to effect the necessary compensation the subsidy of the Government was given not as an addition to the contributions, but as a proportion of the benefits paid. Experience has shown that the deduction from the contributions in respect of women has been too large, or, in other words, the balance of the contributions, after the sinking fund deduction has been made, has proved to be too small to meet the current payments of benefit. The result would have been that approved societies with female members would, generally speaking, have been found to have deficiencies upon the first valuation. It was this state of affairs that the Insurance Commissioners desired, if possible, to avoid. The method adopted in the Bill before Parliament for meeting the situation is, briefly, to reduce the amount of the deduction from the contributions for sinking fund purposes, and thereby to set free a larger sum out of which the current benefits can be paid.

The proposal is to reduce the deduction from 1½d. to 1⅛d., and besides this, it is proposed that, instead of applying the whole of the sum retained out of the contributions towards the liquidation of the "reserve values," the Insurance Commissioners shall apply certain portions thereof to two special funds to be known respectively as the "Contingencies Fund" and the "Special Risks Fund." In the case of women the provision to be made for these funds together amounts to ¼d., so that it will be seen that the sum available for sinking fund purposes will be reduced from 1½d. to ⅞d. The natural consequence will be that the time taken to liquidate the "reserve values" will be much longer than that originally estimated, with the result that for a period of more than thirty years the State contribution to national insurance will be paying for the benefits given at the inception of the Act to persons above age sixteen in excess of the value of their own contributions. This being so, it will be seen that a full generation of new entrants into insurance will receive no direct benefit from the State contributions to the insurance funds.

### A Further State Subsidy.

A determined fight was made by certain ardent advocates of national insurance against such a proceeding as has been indicated above, but the only alternative to the proposals would have involved further large subsidies from the State, and in the present circumstances of national finance the Government decided that it was impossible to meet such a demand.

The financial provisions of the Bill also propose to set up a Women's Equalisation Fund, the purpose of which is to equalise the charges for benefits as between different approved societies insuring women, on account of the observed experience that the sickness claims of married women are higher than those of unmarried women. The limit placed

* Bill 97. Government Publishers. 6d.

by the Bill upon this fund is that the grants made therefrom shall not exceed eight shillings per annum in respect of each of the total number of married women who are members of approved societies. The moneys required for the purposes of the Women's Equalisation Fund are to be provided out of Parliamentary votes specially earmarked, the estimated annual charge under the clause being approximately £250,000.

Part I. of the Bill also provides for the application of the Contingencies Fund and the Special Risks Fund, and for their use in connection with valuation deficiencies. Except as otherwise expressly provided (namely, in connection with the Women's Equalisation Fund), it is proposed that the provisions of the first part of the Bill shall be deemed to have come into operation from the commencement of the principal Act. The object is, of course, to make as good a showing as possible when the first valuations are made; but it is clear that a large amount of adjusting entries will have to be made in the accounts already presented and audited for the past five years, and the officials of approved societies can look forward to having a very busy time on their accounts for some while to come.

In view of certain opposition to the procedure set forth in the Bill as regards the "Special Risks Fund," it was indicated by Sir Edwin Cornwall that the Fund will probably be renamed the "Central Fund," and some slight modifications are to be expected in its constitution during the Committee stage.

### The Simplification Proposals.

The Bill makes restrictions upon the class known as voluntary contributors, limiting all future entries solely to persons who have previously been employed contributors for a period of at least two years. Provision is also made for exempting from insurance persons who are only employed intermittently. These are great improvements. Another very important simplification will arise from the requirement set out in Clause 9, whereby it will in future be necessary for employers who pay low wages to stamp the cards of their employees at the rates applicable to those earning full wages, and if they desire to be recompensed they will have to make a claim on the Insurance Commissioners.

Under Clause 12 some important amendments are made with regard to sickness benefit. In the first place it is proposed to reduce the rate of benefit to 6s. a week for men and to 5s. a week for women if the sickness occurs before 104 weekly contributions have been paid. Secondly, the clause gives precise directions as to the notice to be given to approved societies of the sickness in respect of which a claim for benefit is made. Briefly, if notice is not given within three days of the commencement of sickness, then the benefit shall not be paid except as from the day following that upon which notice is given. This is a very salutary provision, and will enable approved societies to supervise their sickness claims much more thoroughly than has been possible in the past.

More important still, however, is the fact that under this clause the rate of sickness benefit for all future entrants into insurance will be the full ordinary rate (modified as stated above), irrespective of age at entry, and that approved societies will no longer have the power of reducing the rate of benefit when the normal rate is more than two-thirds the amount of wages usually earned. The Bill goes on to provide for the case of persons ceasing to be insured, with the rights, liabilities, and duties of membership of approved societies, and of the procedure regarding transfer from one society to another. Space will not allow us to enter into details. The complicated question of arrears of contributions is also dealt with, but in this matter the proposals of the Bill are not very illuminating, as so much is left to be worked out in the form of regulations to be made by the Insurance Commissioners. It may be said, however, that there is every reason to expect that when the regulations eventually appear they will deal with the matter in a much more practical manner.

### Payments for Inmates of Hospitals.

The section in the principal Act dealing with the payment of benefits in respect of persons who are inmates of hospitals is also to be amended. Curiously enough this clause (clause 20), although one of the longest in the Bill, seems to have entirely escaped attention in the debate. The principle involved is much the same as under the principal Act, for it is only in the last resort, when the insured person has no dependants and when, with his authority, any expenses for which he may be or may become liable, otherwise than to the institution while he is an inmate, have been satisfied, that the balance (if any) of the sickness benefit may, at the discretion of the approved society or insurance committee, be paid in whole or in part to the institution of which he is an inmate. Thus payment for work done on behalf of insured persons by the voluntary hospitals to be derived from insurance funds is farther off than ever. Further, we feel quite certain that the smooth working of the National Insurance Act will never be brought about if a permissive provision on the part of approved societies forms part of the Act. Institutions should give this important matter their immediate consideration with a view to the deletion of this permissive provision and the substitution for it of power to make the provisions of the Act in their relation to institutions not permissive but obligatory on the part of approved societies.

### The Aliens Clause.

Only one clause appears to have been seriously opposed, namely, that which would give to aliens the same benefits as those accorded to British subjects. It was inserted on the recommendation of the Ryan Committee, as it was shown that the cost to the State would be almost negligible, and that it was to the advantage of the community that all workers, even though aliens—and, perhaps, enemy aliens—should have proper provision made for them in sickness.

